# Optimal facial regions for remote heart rate measurement during physical and cognitive activities

**DOI:** 10.1038/s44325-024-00033-7

**Published:** 2024-11-29

**Authors:** Shuo Li, Mohamed Elgendi, Carlo Menon

**Affiliations:** 1 Biomedical and Mobile Health Technology Lab, ETH Zurich, 8008 Zurich, Switzerland; 2 Department of Information Technology and Electrical Engineering, ETH Zurich, 8092 Zurich, Switzerland; 3 Department of Biomedical Engineering and Biotechnology, Khalifa University of Science and Technology, Abu Dhabi, UAE; 4 Healthcare Engineering Innovation Group (HEIG), Khalifa University of Science and Technology, Abu Dhabi, UAE

**Keywords:** Blood flow, Cardiology, Diseases, Health care

## Abstract

Remote photoplethysmography (rPPG) has gained prominence as a non-contact and real-time technology for heart rate monitoring. A critical factor in rPPG’s accuracy is the selection of regions of interest (ROI), as it can significantly influence prediction outcomes. Most studies typically use the forehead and cheeks as ROIs, but little research has explored other facial regions or how stable these ROIs are during physical movement and cognitive tasks. In this study, we analyzed 28 facial regions based on anatomical definitions using two mixed datasets derived from three public databases: LGI-PPGI, UBFC-rPPG, and UBFC-Phys. We applied rPPG algorithms such as orthogonal matrix image transformation (OMIT), plane-orthogonal-to-skin (POS), chrominance-based (CHROM), and local group invariance (LGI). Our findings show that the glabella, medial forehead, lateral forehead, malars, and upper nasal dorsum consistently perform well, with the glabella achieving the highest overall evaluation score. These results offer valuable insights for advancing remote heart rate monitoring technologies.

## Introduction

Heart rate (HR) is one of the most fundamental and important signals among various health-related signals of the human body. Variations in HR over time can reflect different aspects of the subject’s states, such as their health condition^1–3^, nervous system function^4,5^, and emotional fluctuations^6,7^. Traditional methods for measuring HR values include electrocardiograph (ECG)^8^ and contact-based photoplethysmography (cPPG)^9^, both of which require direct contact with the skin, leading to inconvenience and higher costs during the measuring process. Therefore, the demand for remote HR monitoring has been growing in practical applications. Remote photoplethysmography (rPPG) technologies emerged in response to this need. In 2008, Verkruysse et al.^10^ first discovered the underlying relationship between subtle changes in skin color and synchronous HR values. They found that in the RGB color space, the green channel always contains the strongest HR-related information. This discovery paved the way for HR measurement using rPPG algorithms, leading subsequent researchers to focus on developing various algorithms to improve the accuracy of remote HR estimation.

Within the entire process of HR measurement using rPPG methods, the selection of regions of interest (ROIs) is a critical step. This step involves selecting skin pixels on the face for each frame and averaging the pixel intensities in the RGB color space. Choosing appropriate skin pixels can significantly enhance the accuracy of HR detection, and several previous studies have designed various evaluation metrics to select high-scoring ROIs^11–14^. Forehead and cheeks are the most commonly used facial regions because they are believed to provide more accurate HR measurement results, although the specific definitions of ROIs vary across different studies^13,15–17^. Moreover, many other facial regions have been rarely used in previous research and their potentials are not ferreted out^18–21^. According to the experiments conducted by Kim et al.^22^, the differences of HR prediction capabilities within different facial regions could be significant. These differences may be related to the anatomical structure of the human face and the size of ROIs.

In real applications of rPPG algorithms, subjects are not always stationary. Subject’s movements in the recording process can introduce motion artifacts into the extracted signals, thereby reducing the HR estimation accuracy. Additionally, these movements may result in performance variations in HR prediction across different facial regions. Haugg et al.^23^ compared the accuracy of HR measurement for the forehead, left cheek, and right cheek under different movements and discovered that different movements may correspond to different optimal facial regions. However, in-depth research in this field, such as a comprehensive division of facial ROIs and regularities of ROI performance, is currently lacking. Besides physical movements, cognitive tasks like pondering specific problems or playing computer games often exist in human-computer interaction (HCI) scenarios. Although cognitive tasks do not involve large-scale body movements, subjects can still have significant HR changes within a short period. These measurement scenarios are closer to reality and worth further exploration compared with an ideal case when the subject’s HR remains stable.

**Fig. 1:**
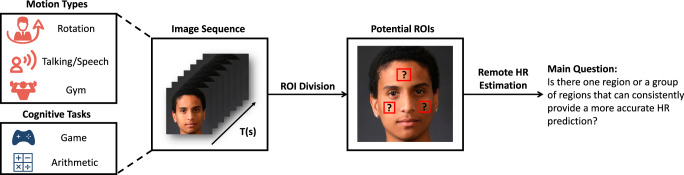
Research focus of this article. Different facial ROIs may have various performances for HR measurement. Forehead and cheeks are frequently used in previous studies, but a systematic and quantitative analysis is still needed. The real human face image was selected from the Real and Fake Face Detection Dataset: https://www.kaggle.com/datasets/ciplab/real-and-fake-face-detection. This dataset is under the CC BY-NC-SA 4.0 License: https://creativecommons.org/licenses/by-nc-sa/4.0/.

In this paper, we defined 28 ROIs of human faces and evaluated the HR measurement performance of each region for four common subject motion types and two cognitive tasks. As is shown in Fig. 1, regarding the ROI performance variation induced by different motion types (e.g., Resting, Rotation, Talking, and Gym) and cognitive tasks (e.g., game and arithmetic), we aim to identify the internal performance consistency. More specifically, we would like to utilize quantitative analyses to determine whether there is one or a group of ROIs that can exhibit excellent performance across different measurement conditions. Research on this aspect can provide deeper insights into the ROI selection process and offer guidance for rPPG technique development in future studies.

## Results

**Fig. 2:**
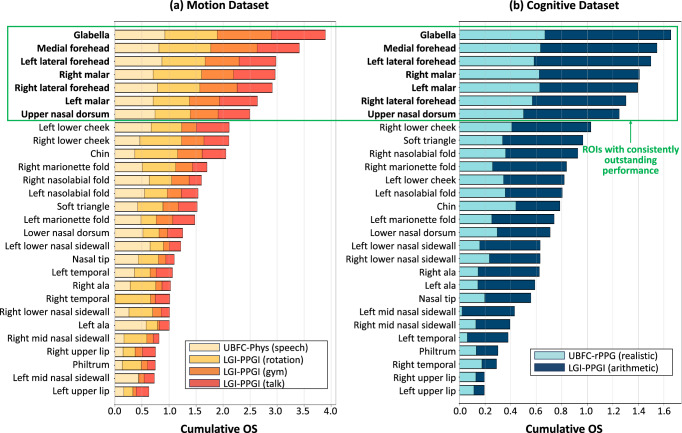
Overall performance evaluation results of the 28 proposed ROIs. Motion Dataset (**a**), Cognitive Dataset (**b**). The results are shown in the form of stacked bar chart. The horizontal axis corresponds to the overall evaluation score (OS) values and the vertical axis corresponds to the names of facial ROIs. The OS of each ROI for all activities is computed based on Equation (4) and stacked together. The facial ROIs are ranked in descending order according to the sum of OS. The regions within the green box are the group of ROIs that have the most outstanding performance.

In the experiments, we visualized the evaluation results of the 28 proposed ROIs on the motion and cognitive datasets respectively which are rearranged from the UBFC-rPPG, UBFC-Phys, and LGI-PPGI databases. The reason for doing this instead of combining all available data is to guarantee the data representativeness in the two general application scenarios of remote HR measurement. We combined three evaluation metrics, MAE, PCC, and SNR, to provide a more comprehensive measurement for HR estimation. To ensure fairness between different activities within the motion or cognitive datasets, each OS was normalized into the range of 0 to 1. In addition, we computed the sum of OS of each facial ROI and ranked them in descending order. The results are depicted using the form of a stacked bar chart in Fig. 2.

From the results in the figure above, it is evident that the glabella achieved the best overall performance on both of the motion and cognitive datasets. In the motion dataset, the sum of OS of the glabella is close to 4.0, which is the theoretical maximum value. In comparison, the performance of the medial forehead was ranked in second place. However, the performance gap was close to 0.5. The green bounding box in Fig. 2 shows a significant inter-group performance variation that the ROIs within the green box had much higher OS compared with other regions. The regions with excellent HR estimation performance are: glabella, medial forehead, left lateral forehead, right lateral forehead, left malar, right malar, and upper nasal dorsum. The remarkable thing is that the glabella and upper nasal dorsum were seldom explored in previous studies. The other five ROIs can be classified into the area of forehead and cheeks which are in constant use. It is worth noting that the performance of left lower cheek and right lower cheek is not very ideal. They were ranked at the eighth and ninth places in the motion dataset. For the cognitive dataset, the left lower cheek didn’t even reach the top-ten regions.

**Fig. 3:**
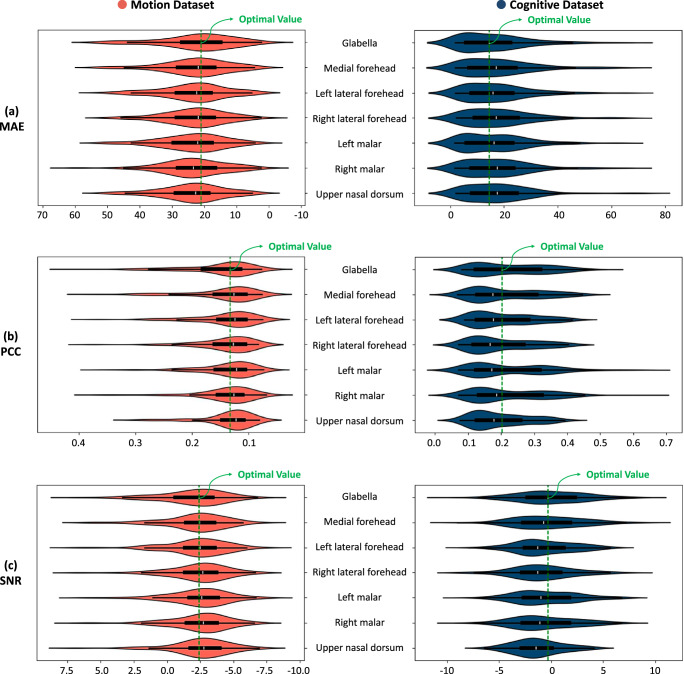
Performance comparison based on individual evaluation metrics. MAE (**a**), PCC (**b**), SNR (**c**). These metrics are defined in Equation (1), (2), and (3). The results are shown in the form of violin plot. The horizontal axis corresponds to the metric values and the vertical axis corresponds to the names of facial ROIs. The median metric values of each ROI are compared and the optimal values are marked using green arrows.

So far, the group of ROIs with the most outstanding overall capabilities for HR estimation has been identified. However, an in-depth performance comparison based on individual evaluation metrics is needed. In Fig. 3, we compared the MAE, PCC, and SNR metrics of these regions in the form of violin plot. The computational formulas are given in Equation (1), (2), and (3).

From Fig. 3, it can be observed that the glabella, which has the highest OS on both of the motion and cognitive datasets, consistently exhibits excellent performance for each individual evaluation metric. The median metric values of the glabella secured the first position in the MAE, PCC, and SNR metrics on both datasets. The PCC and SNR metrics show that the glabella can help extract rPPG signals with higher quality compared with other six regions. The MAE metric demonstrates that the final HR estimation accuracy of the glabella is also in the first place. All of these indicate that the glabella has the potential to be more robust to motion artifacts induced by subject’s movements and also cognitive tasks with less body movements. In short, the glabella demonstrated a significant performance advantage among these top-seven regions, and this advantage remained relatively stable across different evaluation metrics and datasets.

**Fig. 4:**
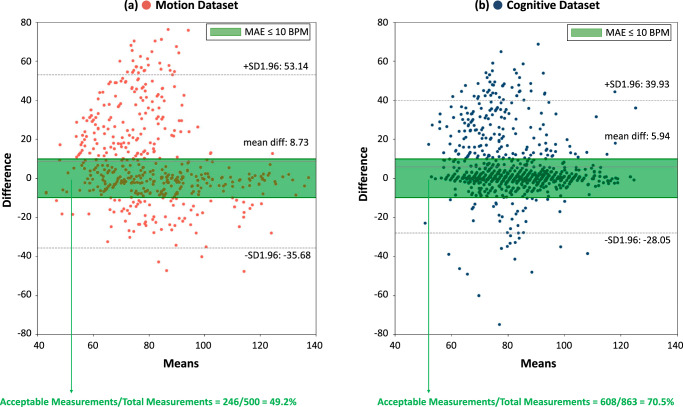
Bland-Altman analysis of using glabella as the ROI for remote HR measurement. Motion Dataset (**a**), Cognitive Dataset (**b**). Measurements within the green range (MAE < = 10 BPM) are regarded as acceptable^24^.

**Fig. 5:**
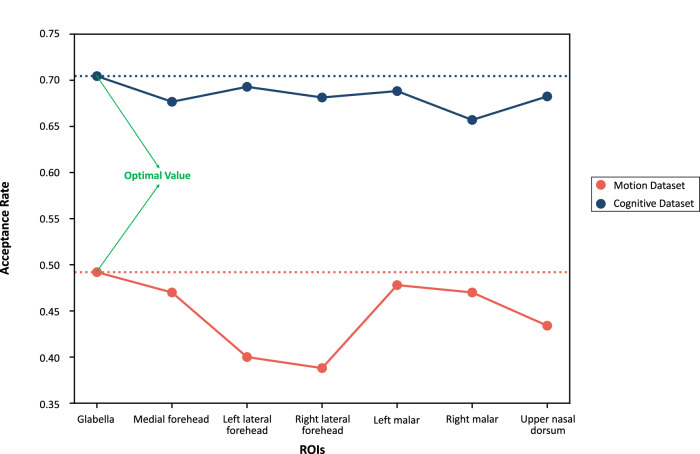
Comparison of acceptance rate across different ROIs. The definition of acceptable measurements is provided in Fig. 4. We define the acceptance rate as the ratio of acceptable measurements to total measurements. The optimal acceptance rates on the motion dataset and cognitive dataset are marked using green arrows.

The Bland-Altman analysis presented in Fig. 4 shows the distribution of the difference between estimated HR and ground truth values in the motion and cognitive datasets when using glabella as the ROI for signal extraction. Each data point in the Bland-Altman plot corresponds to an HR measurement over a time window of 25 s. According to the industrial standard^24^, the measurements with MAEs lower than 10 BPM can be regarded as acceptable. It can be observed that the data points in the cognitive dataset are more centralized towards 0 compared with the condition in the motion dataset. And the proportion of acceptable measurements among total measurements in the cognitive dataset is 70.5%, which is higher than 49.2% in the motion dataset. We define this proportion as the acceptance rate. We also compare the acceptance rate of glabella with other ROIs in Fig. 5. All of these results indicate that the subject’s motions indeed introduce more noise in the measurement process compared to cognitive tasks. And the glabella provides the highest acceptance rate across motion and cognitive datasets.

**Fig. 6:**
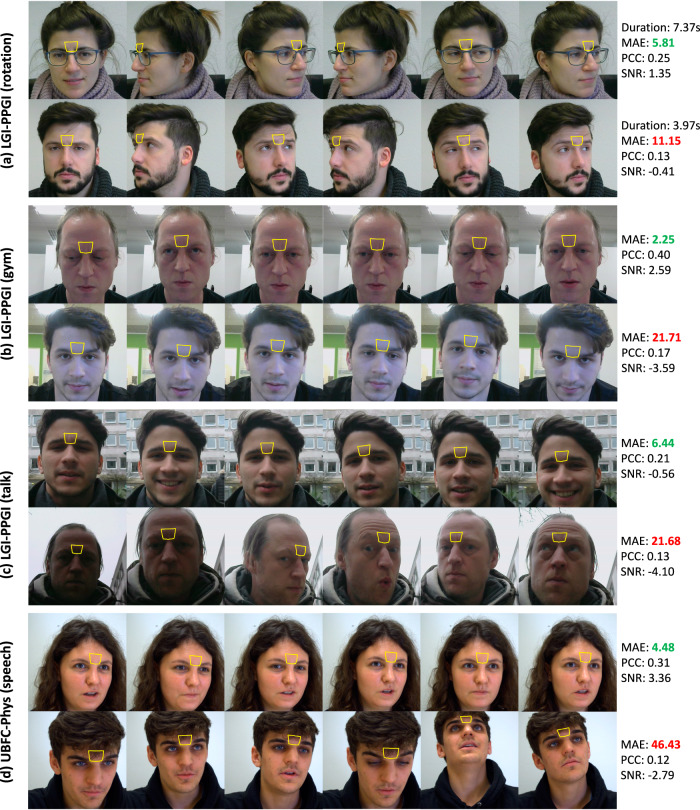
Sample frames of glabella recognition for different states of subjects in the motion dataset. LGI-PPGI (rotation) (**a**), LGI-PPGI (gym) (**b**), LGI-PPGI (talk) (**c**), UBFC-Phys (speech) (**d**). The yellow polygons correspond to the glabella detection of each subject. These polygons were generated based on the selected facial keypoints in Table 1. The performance metrics are presented in the right side. The LGI-PPGI and UBFC-Phys datasets are all under the Creative Commons Attribution 4.0 License: https://creativecommons.org/licenses/by/4.0/.

**Fig. 7:**
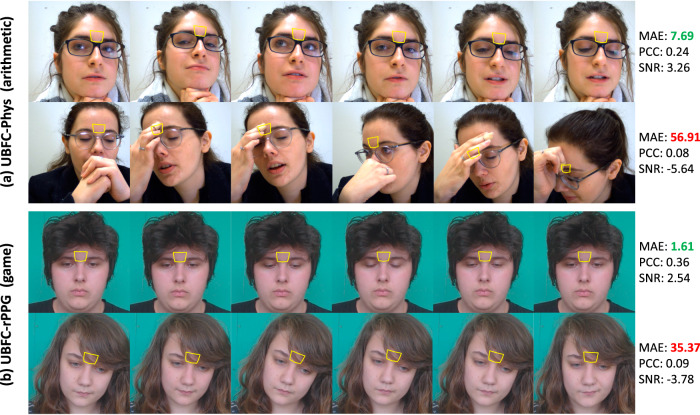
Sample frames of glabella recognition for different states of subjects in the cognitive dataset. UBFC-Phys (arithmetic) **a**, UBFC-rPPG (game) (**b**). The yellow polygons correspond to the glabella detection of each subject. These polygons were generated based on the selected facial keypoints in Table 1. The performance metrics are presented in the right side. The UBFC-Phys dataset is under the Creative Commons Attribution 4.0 License: https://creativecommons.org/licenses/by/4.0/. Sample frames of the UBFC-rPPG dataset are captured from subjects 5 and 11 who have agreed to show their images in publications.

In order to further demonstrate the advantage of extracting rPPG signals from the glabella, we captured some sample frames of participants from videos in the motion and cognitive datasets. In Fig. 6 and 7, we presented sample frames of sub-datasets in the motion and cognitive datasets together with the corresponding performance metrics, and marked the detected glabella using yellow polygons. For each sub-dataset, we provided one positive sample (MAE lower than 10 BPM) and one negative sample (MAE higher than 10 BPM). For the rotation activity in LGI-PPGI, we also presented the duration of performing the same amplitude movement. It can be observed that, regardless of the types of motion, the glabella can be consistently detected. In contrast, the detection of other regions is not as stable. For instance, participants engaged in large motions (e.g., shaking their heads) might have their lateral forehead, malars, and lower cheeks partially or completely obscured. Participants with obvious facial expressions may experience significant deformation in the forehead, malars, and lower cheeks, potentially affecting the SNR of the extracted RGB traces. In comparison, selecting skin pixels from the glabella remains relatively stable in the presence of large motions, facial expressions, and hair occlusion. Nevertheless, there are still many potential factors that can affect the accuracy of HR measurement. In Fig. 6a, the negative sample exhibits much faster head rotation compared to the positive sample. In Fig. 6b, the background lighting might influence the skin tone and thus introduce illumination noises. The negative sample in Fig. 6c contains inconsistent illumination and more significant movements. Besides, the measurement accuracy can drop significantly when the glabella is partially covered. Fig. 6d and 7b show the conditions of the glabella covered by hair. And Fig. 7a shows the example of glabella detection influenced by hand actions.

## Discussion

ROI selection is a critical step that can exert a significant impact on the accuracy of rPPG algorithms in the remote HR monitoring process. There are various factors may contribute to the performance variations in HR prediction capabilities among different facial regions. First, the anatomical structure of human face is highly intricate, with uneven surface topography and variations in dermal and epidermal thickness^22,25^, as well as a complex distribution of the underneath blood vessels. In addition, factors like motion artifacts^11,26^, varying light intensities^27,28^, and potential occlusions^29^ can also influence the algorithm performance. Existing literature has shown a broad array of methods to select skin pixels for extracting high-quality rPPG signals^11–13,29^, but a widely accepted approach is currently lacking. The forehead and cheeks are conventionally regarded as preferred facial ROIs, but their specific definitions vary across different studies.

In contrast to introducing novel rPPG algorithms, our research aimed to compare the performance differences of various facial regions in remote HR prediction. The significance of this research lies in identifying one or a set of facial regions that consistently exhibit superior performance under potential activities in real-world applications of rPPG algorithms. Discovering such patterns can provide valuable insights for future research and practical applications of rPPG methods.

In this study, we systematically defined and analyzed 28 specific facial ROIs, conducting comprehensive investigations on the motion and cognitive datasets based on three public databases: LGI-PGI^30^, UBFC-rPPG^31^, and UBFC-Phys^32^. Based on the results obtained with the combination of MAE, PCC, and SNR metrics, seven regions displayed overall outstanding performance. Among them, the medial forehead, lateral forehead, and malars are more frequently used ROIs in previous research^18–21^, while the glabella and upper nasal dorsum can be viewed as relatively less involved regions. The poorer performance of the remaining regions implies that these areas play a lesser role in the HR monitoring process. Most intriguingly, the glabella consistently exhibited a remarkable performance in the overall evaluation metric. This is contradictory to the traditional notion established in former studies. While the commonly used forehead and malars have shown good performance in our experiments, the glabella, which performed optimally, has been seldom used before. These findings suggest that in many real-world scenarios, the researchers can focus on selecting these seven regions to extract high-quality rPPG signals, with the glabella being a promising candidate for the most suitable ROI.

Furthermore, the glabella possesses several additional advantages. For subjects with prominent fringe, their medial forehead and lateral forehead may be covered due to hair occlusion, thus the SNR of the extracted signals might be significantly reduced. By contrast, the glabella tends to experience less interference from hair obstructions. Additionally, for subjects wearing masks, their malars might be entirely covered, whereas the glabella usually falls outside the coverage area of masks. These advantages provide further support for the practicality and superiority of the glabella as a universally optimal ROI for remote HR measurement.

Nonetheless, our study still has several limitations. It mainly conducted quantitative analyses from a comparative experimental perspective without offering a foundational explanation for the performance variations among different ROIs. In addition to the anatomical differences, Wong et al.^33^ explained the reasons for ROI selection leading to varying HR measurement results from the perspective of facial surface orientation and its impact on diffuse reflection. They discovered that regions facing the recording camera directly can reflect more diffuse components of light, which contain more HR-related information. However, these studies still exhibit limitations in terms of generality and comprehensiveness. We suggest the following potential future research directions in this field:

1. Consider the impact of environmental factors in the ROI selection process of remote HR measurement from different aspects, such as uneven light distribution, hair and beard occlusion, diversity of skin color, etc.
2. Establish a theoretical model that incorporates multiple influencing factors to explain the performance variations among different facial regions. This can help researchers have a deeper understanding of the ROI selection process.
3. In consideration of the optimal facial ROI may vary in different environments, the improvement of current ROI selection methods is necessary. Analyzing existing ROI selection methods and summarizing them to propose more robust metrics can be beneficial for improving the HR estimation accuracy.

## Methodology

### Dataset

In order to ensure the comprehensiveness and reliability of the datasets we used in the experiment, we employed three public datasets for performance evaluation: LGI-PPGI^30^, UBFC-rPPG^31^, and UBFC-Phys^32^. The information is given as follows.

#### LGI-PPGI

This dataset provides four different scenarios simulating the practical applications of rPPG algorithms for HR measurement. These four recording scenarios correspond to four common subject’s motion types. The settings for video acquisition are:

1. Resting: No head and facial motions, static indoor illumination.
2. Rotation: Conversation including head and facial motions, natural varying illumination.
3. Talking: Rotating head, static indoor illumination.
4. Gym: Exercising on a bicycle ergometer, static indoor illumination.

The ground truth cardiac signal is recorded synchronously using fingertip oximeters and provided in the form of cPPG signals and HR values with a frame rate of 60 Hz. The recording camera is Logitech C270 HD with a frame rate of 25 frames per second (FPS) and a resolution of 640 × 480. The total number of participants is six, including five men and one woman.

#### UBFC-rPPG

In 2017, the UBFC-rPPG dataset was proposed together with an unsupervised skin tissue segmentation technique^31^ developed by Bobbia et al. This dataset is also a public database which comprises two sub-datasets. The first sub-dataset is composed of 8 videos with steady participants in an indoor office setting. In the second sub-dataset, participants are required to sit 1 meter in front of the computer and play games to simulate the real HCI scenario. The second sub-dataset contains the data of 42 participants. All videos are recorded by a low-cost webcam (Logitech C920 HD Pro) with the resolution of 640 × 480 and frame rate of 30 FPS. A finger clip sensor is used to record the synchronous cPPG and HR signals.

#### UBFC-Phys

The UBFC-Phys dataset^32^ was collected as an extension of UBFC-rPPG. This dataset is mainly designed for physiological studies under social stress. All of the 56 participants are asked to sit in front of the camera to record facial videos in three stages: rest, speech, and arithmetic. The distance between the participant and the camera is also 1 meter, which is consistent with the UBFC-rPPG dataset. The videos are recorded by an EO-23121C RGB digital camera. The frame rate is 35 FPS and the resolution is of 1024 × 1024. The ground truth cPPG signals are acquired using an electronic wristband.

In our experiment, we rearranged these three datasets into two groups of data. The first group is used for performance evaluation of different subject’s motion types. It contains the rotation, talking, and gym conditions in the LGI-PPGI dataset and the speech condition in the UBFC-Phys dataset. We refer to this set of data as the “motion dataset”. The second group is collected for cognitive tasks which contain the second sub-dataset of UBFC-rPPG and the arithmetic condition in the UBFC-Phys dataset. We call this group of data as the “cognitive dataset”.

### ROI division

**Fig. 8:**
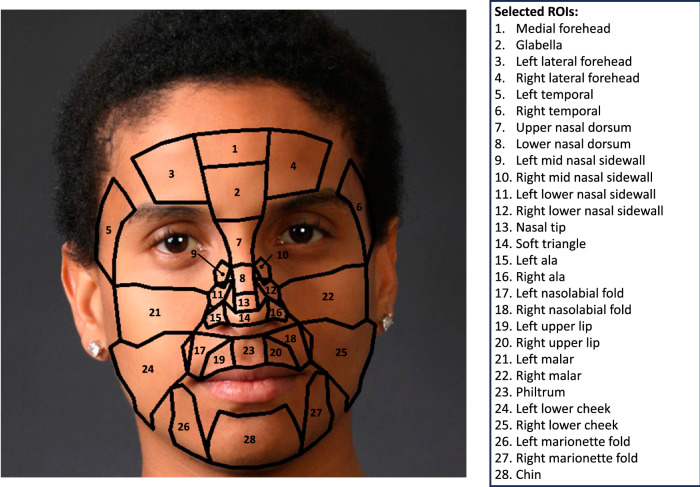
The 28 facial ROIs that have been defined in our study. The definition of ROIs was based on the two-dimensional keypoints extracted by MediaPipe Face Mesh^34,35^. The real human face image was selected from the Real and Fake Face Detection Dataset: https://www.kaggle.com/datasets/ciplab/real-and-fake-face-detection. This dataset is under the CC BY-NC-SA 4.0 License: https://creativecommons.org/licenses/by-nc-sa/4.0/.

In most studies that are relevant to the development of rPPG algorithms, the definition of particular facial ROIs is often unclear. Although many articles have pointed out that regions within or near the forehead and cheeks might possess relatively strong HR-related signals^13,15–17^, there are no standardized methods to define specific facial regions. To clarify this definition, we defined 28 ROIs from an anatomical perspective based on a previous study^25^. These regions cover most of the skin pixels on a human face that can be used for cardiac signal extraction. We defined the keypoint list for each facial ROI using the two-dimensional coordinates and indices of the 468 key points provided by MediaPipe Face Mesh^34,35^. The relevant data has been listed in Table 1. In addition, we provided a sample of ROI division on a virtual human face image in Fig. 8, which illustrates the correspondence between facial ROIs and their names.

**Table 1:** Keypoint list of 28 facial ROIs

ROI Index (k)	ROI Name	Keypoint List
1	medial forehead	10, 109, 108, 151, 337, 338
2	left lateral forehead	67, 103, 104, 105, 66, 107, 108, 109
3	right lateral forehead	297, 338, 337, 336, 296, 334, 333, 332
4	glabella	151, 108, 107, 55, 8, 285, 336, 337
5	upper nasal dorsum	8, 55, 193, 122, 196, 197, 419, 351, 417, 285
6	lower nasal dorsum	197, 196, 3, 51, 5, 281, 248, 419
7	soft triangle	4, 45, 134, 220, 237, 44, 1, 274, 457, 440, 363, 275
8	left ala	134, 131, 49, 102, 64, 219, 218, 237, 220
9	right ala	363, 440, 457, 438, 439, 294, 331, 279, 360
10	nasal tip	5, 51, 45, 4, 275, 281
11	left lower nasal sidewall	3, 217, 126, 209, 131, 134
12	right lower nasal sidewall	248, 363, 360, 429, 355, 437
13	left mid nasal sidewall	188, 114, 217, 236, 196
14	right mid nasal sidewall	412, 419, 456, 437, 343
15	philtrum	2, 97, 167, 37, 0, 267, 393, 326
16	left upper lip	97, 165, 185, 40, 39, 37, 167
17	right upper lip	326, 393, 267, 269, 270, 409, 391
18	left nasolabial fold	97, 98, 203, 186, 185, 165
19	right nasolabial fold	326, 391, 409, 410, 423, 327
20	left temporal	54, 21, 162, 127, 116, 143, 156, 70, 63, 68
21	right temporal	284, 298, 293, 300, 383, 372, 345, 356, 389, 251
22	left malar	126, 100, 118, 117, 116, 123, 147, 187, 205, 203, 129, 209
23	right malar	355, 429, 358, 423, 425, 411, 376, 352, 345, 346, 347, 329
24	left lower cheek	203, 205, 187, 147, 177, 215, 138, 172, 136, 135, 212, 186, 206
25	right lower cheek	423, 426, 410, 432, 364, 365, 397, 367, 435, 401, 376, 411, 425
26	chin	18, 83, 182, 194, 32, 140, 176, 148, 152, 377, 400, 369, 262, 418, 406, 313
27	left marionette fold	57, 212, 210, 169, 150, 149, 176, 140, 204, 43
28	right marionette fold	287, 273, 424, 369, 400, 378, 379, 394, 430, 432

The keypoint lists were defined according to the face features provided by MediaPipe Face Mesh^34,35^ which contains 468 keypoints. The keypoint list of each ROI was utilized to define the polygons that can enclose the selected skin pixels.

### HR estimation pipeline

**Fig. 9:**
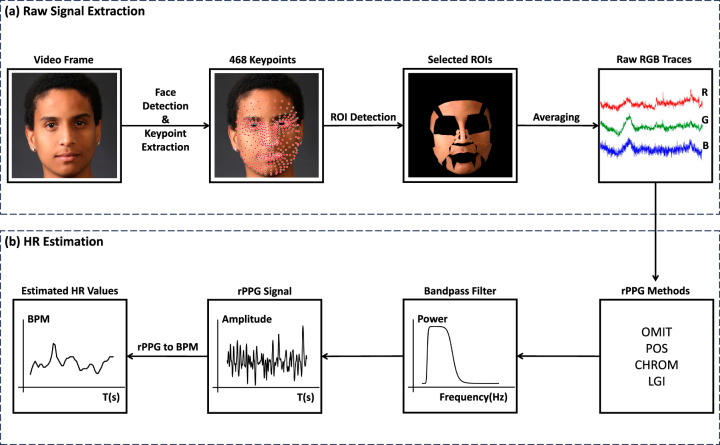
The general pipeline of using rPPG algorithms for HR estimation from facial videos. The whole workflow is comprised of two phases: Raw Signal Extraction (**a**) and HR Estimation (**b**). Note that HR = heart rate, Raw RGB Traces = time series of average pixel intensities across red, green, and blue channels, rPPG Methods = selected algorithms for converting RGB traces into remote photoplethysmography signals, BPM beats per minute, OMIT orthogonal matrix image transformation, POS plane-orthogonal-to-skin, CHROM the chrominance-based method, LGI local group invariance. The real human face image was selected from the Real and Fake Face Detection Dataset: https://www.kaggle.com/datasets/ciplab/real-and-fake-face-detection. This dataset is under the CC BY-NC-SA 4.0 License: https://creativecommons.org/licenses/by-nc-sa/4.0/.

Figure 9 illustrates the general workflow employed in this study for extracting HR from RGB facial videos using rPPG methods. The entire process can be divided into two main components: (a) Raw Signal Extraction, and (b) HR Estimation. The reason for doing this division is that we would like to estimate HR values in an offline fashion, thus avoiding redundant RGB signal extraction and thereby conserving computational resources.

In the “Raw Signal Extraction” phase, we utilized the MediaPipe Face Mesh API^34,35^ to extract facial keypoints from each frame, totaling 468 keypoints. These facial keypoints correspond to specific positions on the detected face, and each keypoint is uniquely identified by a serial number ranging from 0 to 467. The position of each keypoint is denoted as (x, y, z), where x and y represent the 2D coordinates of the keypoint within the current frame, and z represents the depth information. Subsequent to the extraction of facial keypoints, we divided each frame’s facial region based on the 28 pre-defined ROIs, each associated with a set of keypoint serial numbers. In the next step, we computed the average pixel intensities for each ROI across the RGB channels respectively, resulting in the generation of raw RGB traces. In short, in the “Raw Signal Extraction" part, the input consists of a facial video sequence, and the output comprises 28 raw RGB traces of equal duration.

The “HR Estimation" phase is applied to convert the previously extracted RGB traces into estimated HR values. Although early research^10^ has shown that the green channel contains more pulse information, the HR measurement error can be sometimes relatively high when only using the green channel for estimation. Therefore researchers invented different algorithms to convert RGB signals into HR signals in a more accurate way. Based on a former comparative study of traditional rPPG algorithms, orthogonal matrix image transformation (OMIT)^36^, plane-orthogonal-to-skin (POS)^37^, the chrominance-based method (CHROM)^38^, and local group invariance (LGI)^30,39^, have demonstrated superior overall performance.^23^ We selected them as the rPPG algorithms for converting RGB traces to PPG signals in our experiments. The basic principles of the selected rPPG algorithms are introduced in Table 2. To improve the signal to noise ratio (SNR), a sixth-order Butterworth bandpass filter^40,41^ with the cut-off frequencies of 0.65 Hz and 4 Hz was applied for noise removal. For the resulting rPPG signals, Welch’s method^42^, which is based on power spectrum density (PSD) estimation, was leveraged to compute HR values over a sliding window with a length of 6 seconds and overlap between windows of 5 seconds. This entire phase was implemented in the pyVHR framework^43,44^, which is primarily used for the development, evaluation, and statistical analysis of commonly used rPPG algorithms.

**Table 2:** Summary of the selected rPPG methods

Publication Year	Authors	Method Name	Description
2013	De Haan et al.^38^	CHROM	In order to reduce the influence of the subject’s motion, the chrominance-based (CHROM) method does not use the original pixel values of RGB channels. Instead, this algorithm calculates the temporal average on each color channel and then combines different color channels after normalization to extract pulse signals.
2017	Wang et al.^48^	POS	The Plane-Orthogonal-to-Skin (POS) method is based on a dichromatic model. It projects the normalized RGB signal onto the plane which is orthogonal to the skin-tone and uses alpha-tuning to find the exact projection direction.
2018	Pilz et al.^30^	LGI	The Local Group Invariance (LGI) method uses a differentiable local group to define a group of invariant features and create feature space. The HR signals are constructed dynamically using covariance matrix, stochastic frequency representation, and recursive inference.
2022	Casado et al.^36^	OMIT	The thin QR decomposition is computed based on the RGB matrix using Householder Reflections. Then the projection matrix is calculated. The target PPG signal is extracted based on the projection matrix through orthogonal projection.

These methods are ranked according to the published time.

### Evaluation metric

In order to determine the performance of each facial ROI for HR measurement, we employed three evaluation metrics: mean absolute error (MAE), Pearson correlation coefficient (PCC), and signal-to-noise ratio (SNR)^45–47^. The MAE metric is widely used in the process for accuracy analysis. The formula for the MAE metric is as follows:

1 $${\rm{MAE}}=\frac{1}{m}\mathop{\sum }\limits_{i=1}^{m}| {\hat{h}}_{i}-{h}_{i}|$$

where *m* is the total number of sample points, *i* represents the current sample point, $\widehat{H}$ denotes the estimated HR sequence, and *H* denotes the ground truth HR sequence.

Using MAE alone for evaluation may not provide a comprehensive assessment of the results. Therefore, we also utilized the PCC metric. Unlike MAE, PCC is calculated based on the rPPG signals and does not directly measure the accuracy of HR estimation. PCC is used to quantify the linear dependence relationship, comparing the trends in waveform variations and expressing the correlation with a value ranging between -1 and 1. Computing the PCC metrics on long time series might lead to potential noise, so we included a windowing operation to segment the rPPG signals. The calculation formula for the PCC metric is shown as follows:

2 $${\rm{PCC}}=\frac{1}{n}\mathop{\sum }\limits_{j=1}^{n}\frac{m\mathop{\sum }\nolimits_{i = 1}^{m}{X}_{j}\left[i\right]{Y}_{j}\left[i\right]-\mathop{\sum }\nolimits_{i = 1}^{m}{X}_{j}\left[i\right]\mathop{\sum }\nolimits_{i = 1}^{m}{Y}_{j}\left[i\right]}{\sqrt{m\mathop{\sum }\nolimits_{i = 1}^{m}{\left({X}_{j}\left[i\right]\right)}^{2}-{\left(\mathop{\sum }\nolimits_{i = 1}^{m}{X}_{j}\left[i\right]\right)}^{2}}\sqrt{m\mathop{\sum }\nolimits_{i = 1}^{m}{\left({Y}_{j}\left[i\right]\right)}^{2}-{\left(\mathop{\sum }\nolimits_{i = 1}^{m}{Y}_{j}\left[i\right]\right)}^{2}}}$$

where *n* represents the total number of signal segments after the windowing operation, *j* represents the current segment number, *m* denotes the total number of sample points within the segment, *i* denotes the current sample point of the segment, *X* and *Y* represent the estimated rPPG signals and reference cPPG signals, respectively.

The SNR metric is also intensively used in rPPG algorithm development. This metric is evaluated through computing the ratio between the signal intensity and background noise. Compared with MAE and PCC, SNR can assess the relative estimation error under the influence of noises. Formally the metric is computed as:

3 $${\rm{SNR}}=\frac{1}{n}\mathop{\sum }\limits_{j=1}^{n}10\,{\log }_{10}\left(\frac{{\sum }_{V}{\left({{\rm{U}}}_{j}\left[v\right]\cdot {\rm{F}}\left\{{X}_{j}\right\}\left[v\right]\right)}^{2}}{{\sum }_{V}{\left(\left(1-{{\rm{U}}}_{j}\left[v\right]\right)\cdot {\rm{F}}\left\{{X}_{j}\right\}\left[v\right]\right)}^{2}}\right)$$

where *n* and *j* are still the total number of signal segments and the current segment number. ${\rm{F}}\left\{\right\}$ represents the fast Fourier transform (FFT) operation, ${\rm{U}}\left[v\right]$ is a binary mask in the frequency domain that can select the power within ±12 BPM range around the reference HR and its first harmonic, *V* represents the entire frequency space, and *X* denotes the rPPG signals estimated by algorithms.

In order to have a clear data visualization, we constructed an overall evaluation score (OS) based on these two metrics. For each facial ROI, after computing the MAE and PCC metrics, the median of all participants were calculated and saved for the OS evaluation. Meanwhile, in order to ensure a fair comparison of ROI performance across different motion types, we normalized the raw evaluation metrics. The mathematical definition of the OS on one specific video activity is as follows:

4 $${{\rm{OS}}}_{k}=\frac{1}{3}\left(\frac{{{\rm{MAE}}}_{\max }-{{\rm{MAE}}}_{k}}{{{\rm{MAE}}}_{\max }-{{\rm{MAE}}}_{\min }}+\frac{{{\rm{PCC}}}_{k}-{{\rm{PCC}}}_{\min }}{{{\rm{PCC}}}_{\max }-{{\rm{PCC}}}_{\min }}+\frac{{{\rm{SNR}}}_{k}-{{\rm{SNR}}}_{\min }}{{{\rm{SNR}}}_{\max }-{{\rm{SNR}}}_{\min }}\right)$$

where k denotes the current ROI number which was defined in Table 1, $\min$ and $\max$ represent the minimum and maximum of the corresponding metric for all facial ROIs, respectively. The definitions of MAE, PCC, and SNR metrics have been provided in Equation (1), (2), and (3).

It is worth noting that, in cases where the predicted values and actual values have different lengths, we employed linear interpolation to align them.

In this study, our research focus is on exploring different ROIs that are suitable for HR measurement from facial videos. Specifically, we paid attention to the ROIs that are more robust to different subject’s movements and cognitive tasks. To provide a more comprehensive and representative data analysis, we constructed the motion and cognitive datasets from the LGI-PPGI, UBFC-rPPG, and UBFC-Phys databases. We leveraged four best-performing traditional rPPG algorithms, OMIT, POS, CHROM, and LGI, as benchmarks and compared the performance differences within 28 facial ROIs. From the experimental outcomes, the main discoveries can be summarized as follows:

1. We defined 28 facial ROIs for remote HR measurement and provided their exact positions using the keypoint lists based on the MediaPipe Face Mesh API.
2. There are seven regions can exhibit excellent overall performance for remote HR estimation across different motion types. They are: medial forehead, glabella, left lateral forehead, right lateral forehead, left malar, right malar, and upper nasal dorsum.
3. We found that the glabella consistently achieved an outstanding performance compared to other regions. Thus we recommend to use it as the most suitable facial region in future studies of rPPG methods.

To the best of our knowledge, there has been no previous research on the impact of ROI selection on the performance of remote HR monitoring considering different video activities in application scenarios. The regularities we have summarized in our experiments can serve as a reference for future development of rPPG algorithms, including seven facial regions with excellent robustness, among which the glabella exhibits the overall highest ranking. However, this article only conducted a quantitative analysis from an experimental perspective. In future research on the ROI selection process, more theoretical support and physical model construction will be needed, enabling researchers to reach a deeper understanding of this field.

## Data Availability

The LGI-PPGI and UBFC-Phys dataset are under the Creative Commons Attribution 4.0 License: https://creativecommons.org/licenses/by/4.0/. Subject 5 and 11 of the UBFC-rPPG dataset have agreed to show their images in ppt, website, report, and publications. The LGI-PPGI dataset can be accessed at https://github.com/partofthestars/LGI-PPGI-DB. The UBFC-rPPG dataset can be accessed at https://sites.google.com/view/ybenezeth/ubfcrppg. The UBFC-Phys dataset can be accessed at https://sites.google.com/view/ybenezeth/ubfc-phys.

## Code availability

All code used for the experiments is open source with no restrictions and can be found on https://github.com/shuoli199909/optimal_roi_rppg. Code used for each rPPG algorithm is sourced from the pyVHR package at https://github.com/phuselab/pyVHR.
